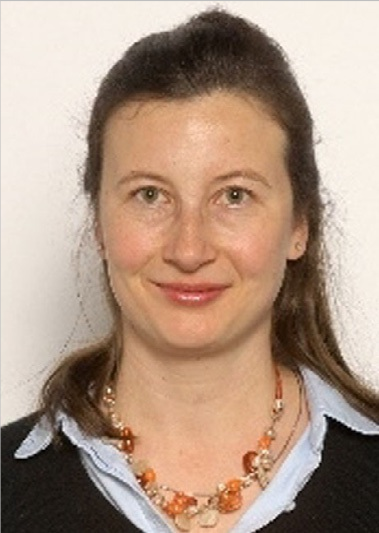# Explorations into the Effect of *meso*‐Substituents in Tricarbocyanine Dyes: A Path to Diverse Biomolecular Probes and Materials

**DOI:** 10.1002/anie.202008075

**Published:** 2020-12-28

**Authors:** Rüdiger M. Exner, Fernando Cortezon‐Tamarit, Sofia I. Pascu

**Affiliations:** ^1^ Department of Chemistry University of Bath Claverton Down Bath BA2 7AY UK

**Keywords:** chemical probes, cyanine, fluorescence, imaging agent, *meso*-substitution

## Abstract

Polymethine cyanine dyes have been widely recognized as promising chemical tools for a range of life science and biomedical applications, such as fluorescent staining of DNA and proteins in gel electrophoresis, fluorescence guided surgery, or as ratiometric probes for probing biochemical pathways. The photophysical properties of such dyes can be tuned through the synthetic modification of the conjugated backbone, for example, by altering aromatic cores or by varying the length of the conjugated polymethine chain. Alternative routes to shaping the absorption, emission, and photostability of dyes of this family are centered around the chemical modifications on the polymethine chain. This Minireview aims to discuss strategies for the introduction of substituents in the meso‐position, their effect on the photophysical properties of these dyes and some structure–activity correlations which could help overcome common limitations in the state of the art in the synthesis.

## Graphical abstract

Tricarbocyanine dyes are widely recognized as highly fluorescent compounds of use in a diverse range of biomedical and technical applications. This Minireview discusses the impact of different *meso*‐substituents and how they may be used in the design of functional probes.

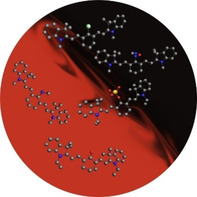

## 1 Introduction

Polymethine cyanine dyes are important fluorescent building blocks commonly used for protein labeling, and cell imaging.^[1]^ The advantages of penta‐ and heptamethine dyes include that their absorption and emission maxima are found in the near‐infrared window of the electromagnetic spectrum (650–1200 nm). In this region, absorption, autofluorescence, and scattering in biological tissue reach local minima.^[2]^ This is especially important when in vivo experiments are performed alongside the imaging of single cells or cell colonies in preclinical investigations: in these contexts, the tissue penetration of excitation beams and emitted light are of paramount importance. Furthermore, structural modifications allowing for orthogonal functionalization may be carried out without significantly affecting their photophysical properties. Thus, bi‐ or tri‐functional cyanine‐based fluorescence emission probes can be prepared without significantly changing their quantum yields, extinction coefficients, or near‐infrared emission.^[3]^

Some derivatives have shown to selectively accumulate in cancer cells and are promising probes in fluorescence‐guided surgery.^[4]^ A monoclonal antibody‐tagged conjugate, bevacizumab‐IR 800CW, used in the fluorescence‐guided surgery of primary breast cancer, passed phase II clinical trials and led to high signal‐to‐background ratios, even at sub‐micromolar concentrations.^[5]^

**Scheme 1:**
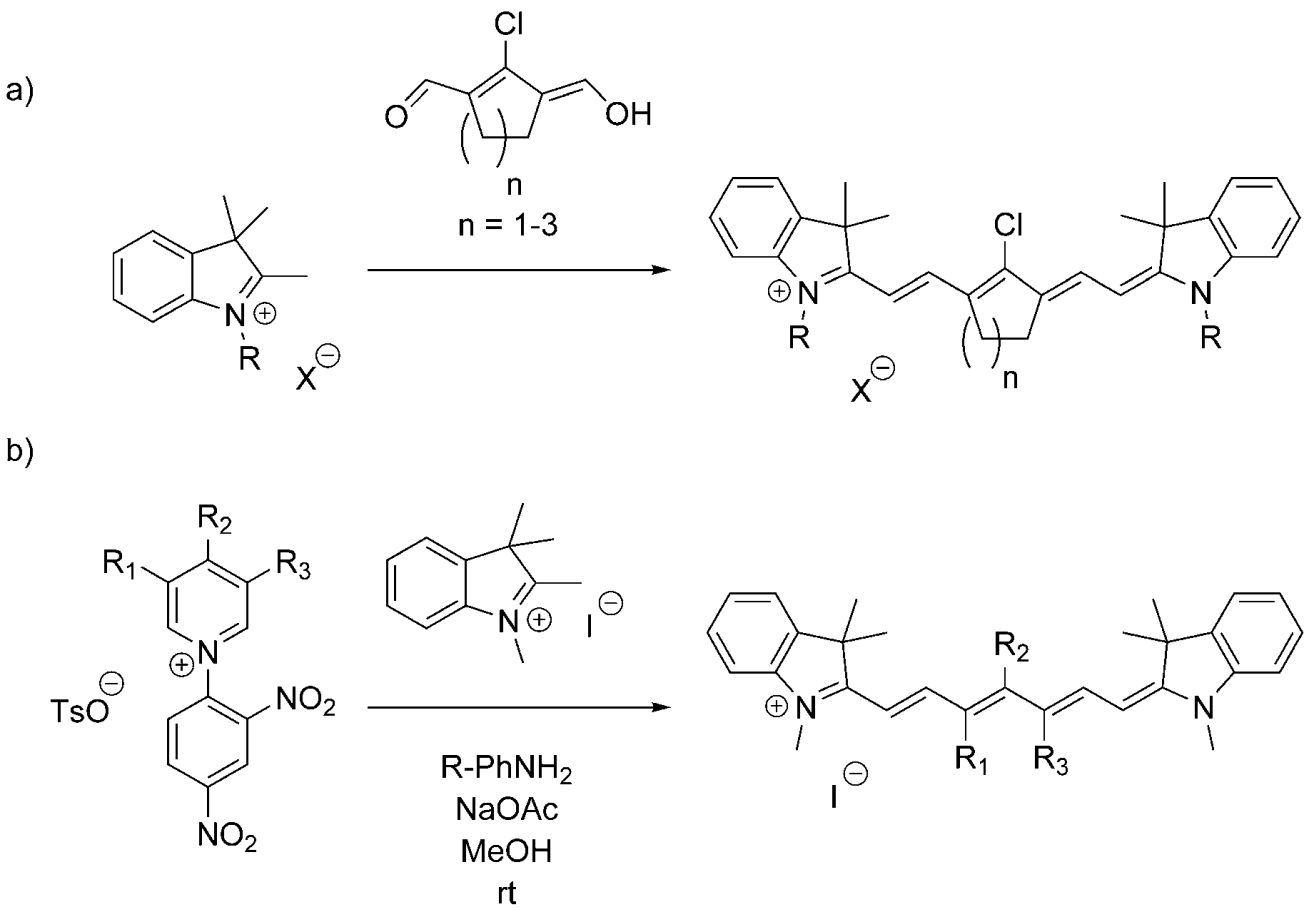
General synthesis of chain‐substituted cyanine dyes via a) condensation or b) ring‐opening of Zincke salts (above), where X=Cl, Br, I; R=alkyl, benzyl; R_1_, R_2_, R_3_=various substituents, see for example ref. [10].

Given the promising results of these highly fluorescent probes, understanding the relationship between structure, pharmacokinetics, and photophysical properties is of great importance in order to fine‐tune absorption/emission, photostability, and in vivo behavior. While early reports on heptamethine cyanine dyes, such as the clinically used indocyanine green (ICG), pointed out that these suffered from low fluorescence quantum yields, unfavorable aggregation, and low photostability, the rigidification of the polymethine chain by introduction of cyclic motifs led to remarkable advances. A new generation of related probes with improved physicochemical properties in living cells thus emerged.^[6]^ The observed improvements were attributed to limiting the radiation‐free loss of energy through rotation and planarization, as well as steric demand. Full conformational restraint of a heptamethine backbone failed to produce any noticeable improvement in brightness, quantum yield, or fluorescence lifetimes.^[7]^ This suggested that photoisomerization of heptamethine cyanines does not play a significant role in the excited‐state behavior of these dyes.^[7]^ Instead, the observed changes may be related to variations in bond angles or lengths, and insight into the structure–activity relationships is necessary. The main strategies for the synthesis of chain‐substituted dyes are shown in Scheme 1. These routes led to structural rigidification through the inclusion of either a central 5‐ or 6‐membered ring, or shielded dyes through bulky substituents, which in turn led to reduced rates of photobleaching and increased kinetic stability of these dyes.

The main synthetic strategy for the rigidification of the polymethine chain is the use of a conjugated dialdehyde containing a cyclic motif. These are typically obtained from a double Vilsmeier–Haack‐type reaction involving a cyclic ketone, such as cyclopentanone, cyclohexanone, or cycloheptanone.[^6b^, ^8^] In the course of this reaction, the ketone is 1,3‐bisformylated and the oxygen substituent is substituted by a chloride. Using phosphoryl bromide, rather than phosphoryl chloride, yields the respective *meso*‐Br derivatives.^[8b]^ Another route to access the dialdehydes is by employing 1‐substituted cyclohexenes as the starting materials.^[6b]^

A *meso*‐Cl cyclohexene motif was reported to offer the highest molecular brightness, compared to the dyes featuring cyclopentene and cycloheptene motifs.^[6b]^ The molecular brightness for the cyclopentene derivative was found to be marginally lower and the cycloheptene derivative was found not to be fluorescent. While absorption maxima are similar for the cyclohexene and cycloheptene derivatives, cyclopentene derivatives display a bathochromic shift (ca. 20 nm).[^6b^, ^8c^] More importantly, the nature of the *meso*‐heteroatom at the center of the cyanine polymethine chains has been the focus of numerous studies investigating the routes to substitution and the effect of this substituent on the photophysical properties of the resulting dye. While most authors report on the synthetic modification of the *meso*‐substituent after the condensation of the aldehyde and the indoleine, it is possible to choose an appropriate precursor, or functionalize it before the condensation, as demonstrated by various authors who performed Suzuki–Miyaura couplings with the bis‐imine.^[9]^

The *meso*‐chloride dyes are known to be reactive in most organic solvents, with the chloride acting both as a nucleofuge and as a leaving group in the palladium‐catalyzed Suzuki–Miyaura or Sonogashira coupling reactions.^[11]^ This has resulted in a plethora of easily accessible, functional dyes with new physicochemical properties. In recent years, the introduction and use of such functional molecules, either as ratiometric or as targeted probes has been investigated. Also, *meso*‐substitution, especially with charged functional groups, drastically affects the pharmacokinetic behavior, distribution, excretion, and toxicity.^[12]^ Discrepancies regarding the photophysical properties within the literature are a significant problem and only a small number of systematic studies intercomparing the properties of various classes of *meso*‐substituted dyes were published.[^11a^, ^13^] This compelled us to explore the structural databases (see Section 2) aiming to ascertain a deeper understanding of their structure–activity relationships.

## 2 Structural Insights

**Figure 1:**
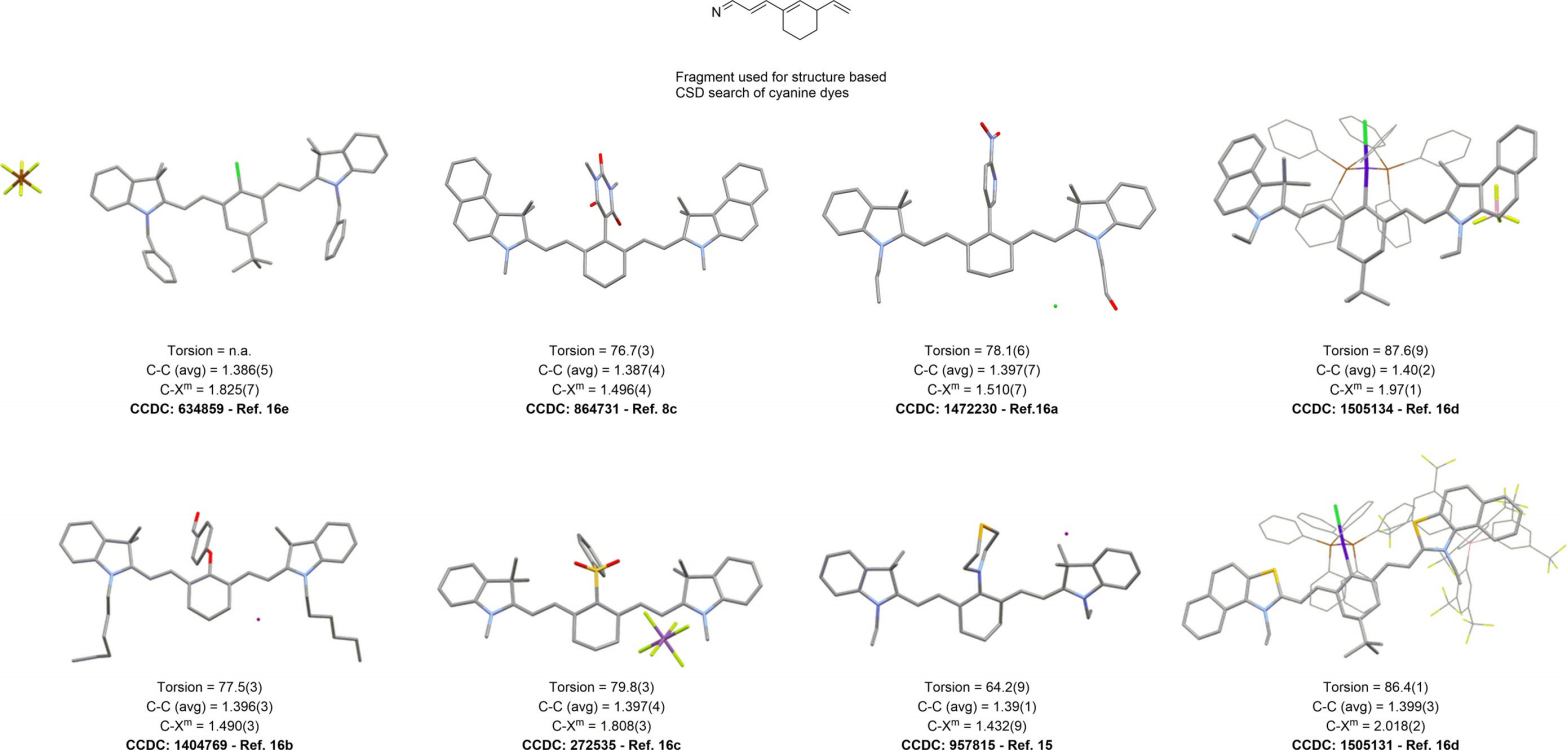
A comparison of selected crystal structures of different substituted cyanine dyes. Torsion angles refer exclusively to the *meso*‐position. Values of angles are in (°), distances are given in Ångström. (Note: due to the simplicity of this fragment, not all CCDC Conquest hits found in the CSD v. 5.41 database, updated 2020 were cyanine dyes.) Therefore, the classic definition was used, according to which cyanine dyes are nitrogen centers connected by conjugated double bonds. Merocyanines were excluded from the CCDC search. After disregarding the compounds which did not fulfil this criterion, polymorphs and different salts of the same basic cyanine dye, a total of 15 unique molecules were selected, most of which featured a simple *meso*‐Cl substituent.)

Whilst a search on Scifinder of “aminocyanine”, “keto‐polymethine” and “*meso* cyanine” yielded 241 unique references (March 2020), we found that the nature of many of these compounds remains to be structurally elucidated. The earliest reports on *meso*‐substituted dyes date back several decades and the focus of this Minireview will be on more recent publications, which detail the photophysical properties or biochemical applications additionally to their unequivocal molecular structure elucidation. The crystal structure mining in the CSD database (using ConQuest^[14]^ CCDC software) employing a simplified cyanine backbone (Figure 1) yielded merely 35 hits. However, a deeper insight into their reported structural parameters revealed that many of these hits show extensive disorder of the cyanine cores and poor quality R‐factors. Figure 1 depicts selected hits and compares the structural parameters of the most representative *meso*‐substituted cyanine dyes. Interestingly, the notable exception is the *meso*‐*N*‐selenomorpholine‐substituted compound which exhibits a smaller dihedral angle by ca. 13°. However, the number of crystal structures is too low to allow for any statistical analysis and deeper data‐mining approaches.^[15]^

## 3 Substitution of meso‐Chloride Substituents

**Scheme 2:**
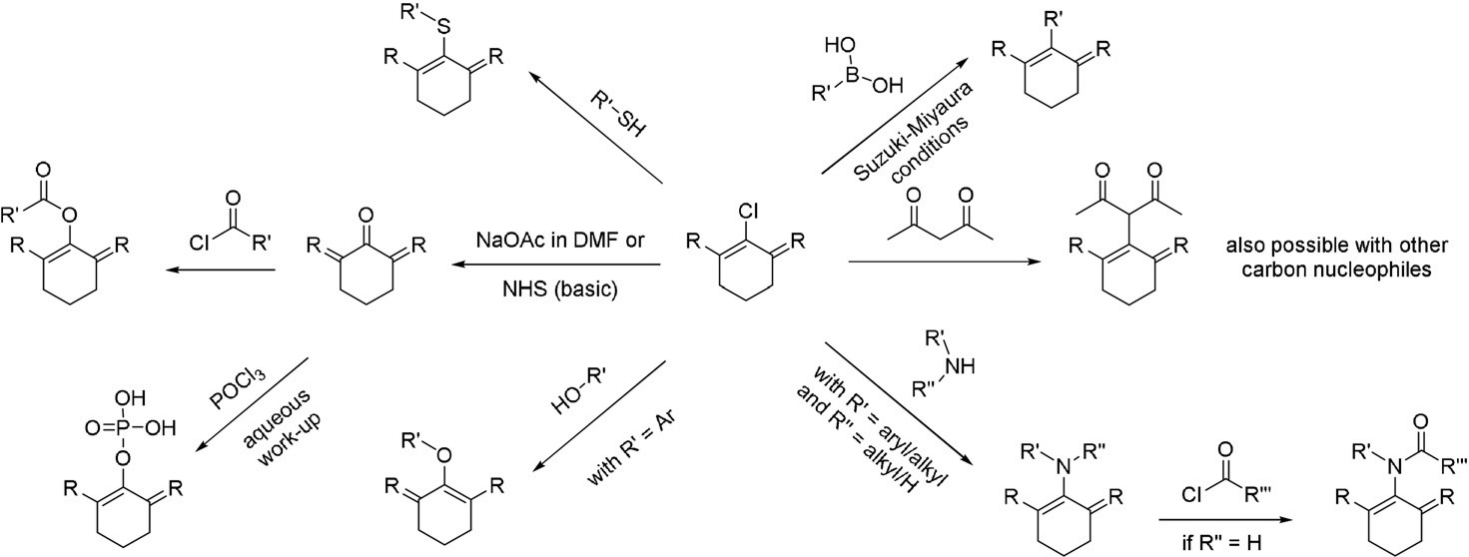
Overview of common reactions to access structurally diverse cyanine imaging probes for biochemical species or processes and agents for photodynamic therapy.

Two routes to dehalogenation of *meso*‐Cl dyes have been described. König et al. described the catalytic dehalogenation using Pd(PPh_3_)_4_ in a mixture of degassed water and degassed DMF at elevated temperatures.^[11a]^ Strekowski et al. on the other hand describe the dehalogenation of *meso*‐Cl, *meso*‐OAr, and *meso*‐SAr with an excess of either sodium ethanethiolate or a mixture of sodium thiophenolate and diphenyl phosphine at elevated temperatures.^[6c]^ Dehalogenated derivatives will not be discussed in this Minireview. Instead, the focus will be on the introduction of carbon‐, nitrogen‐, oxygen‐, and sulfur‐based substituents in the *meso*‐position. A wide variety of such derivatives can be accessed through nucleophilic substitution of the *meso*‐Cl dyes (Scheme 2).

Such reactions probably proceed following an S_RN_1 mechanism. Due to their simplicity, and the often excellent yields, numerous studies using this pathway have been published.[^11b^, ^17^] The observed changes in photophysical properties are remarkable and stretch from increased photostability over direct labeling of proteins to increased Stokes shifts and new functionalities.[^11b^, ^18^] While most of the work has been focusing on the synthesis of *meso*‐nitrogen, ‐oxygen, and ‐sulfur derivatives, the reaction of cyanine dyes with carbon nucleophiles is, so far, rarely found in the literature. While some patents mention the synthesis of nitrile derivatives, full characterization or investigation of such derivatives has not been reported to date, to the best of our knowledge. We hypothesize that this might be due to the fact that side‐reactions limit the accessibility of *meso*‐nitrile derivatives.^[19]^ Likewise, the characterization of other pseudo‐halogenated derivatives has not been encountered in the literature to date, with the notable exception of the *meso*‐azide derivative,[^18a^, ^20^] despite the useful reactivities they may offer. While the syntheses of *meso*‐selenium derivatives^[21]^ and *meso*‐phosphine derivatives^[22]^ have been described as probes for various oxidative species, they will not be discussed here, as their optical properties have not received a rigorous dissemination in the literature. Instead, the focus will be on design strategies of functional substituted probes based on *meso*‐carbon, *meso*‐nitrogen‐, *meso*‐oxygen‐, and *meso*‐thiol‐substituted cyanine dyes.

Numerous studies explore dyes obtained from palladium‐catalyzed cross‐coupling reactions. Crystal structures of Pd‐substituted dyes have been published by Davydenko et al., who demonstrated the suppressive effect of bulky *meso*‐substituents on aggregation.^[16d]^

### 3.1 Introduction of Carbon Substituents

**Scheme 3:**
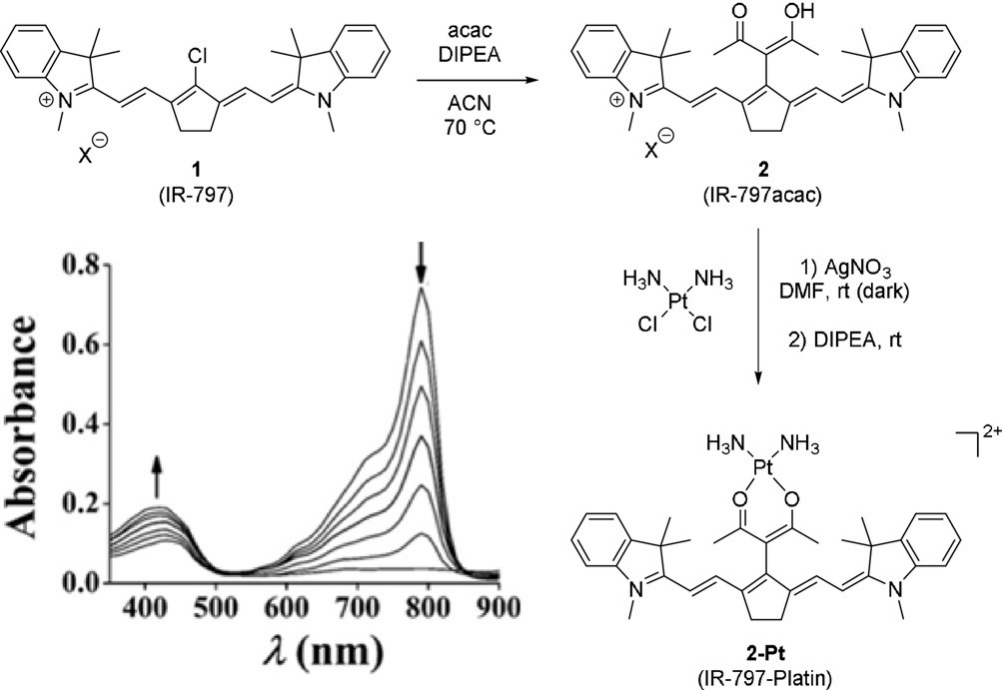
Synthesis of **2** and subsequent formation of the cytotoxic platinum complex **2‐Pt**. Bottom left corner: photodegradation of **2‐Pt**‐Platin, at 30′′ and 1′ intervals. Image used with permission from Ref. [23].

While most carbon‐based substituents are introduced via cross‐coupling reactions, there are three examples of readily available carbon nucleophiles which were successfully reacted with tricarbocyanine scaffolds. The most noteworthy is the reaction with acetylacetone (acac) under basic conditions, as shown by Mitra et al.^[23]^ This modification introduces a ligand into the original structure and was used as a platinum‐containing prodrug for photodynamic therapy (Scheme 3). The absorption maximum of the resulting dye **2** was blue‐shifted by ca. 5 nm. Combined with an emission maximum, red‐shifted by about 5 nm, the Stokes shift is increased by ca. 10 nm. Mitra et al. also reported that, while the quantum yield of **2** is increased compared to **1**, upon binding of diamagnetic Pt^II^, a partial quenching of fluorescence was observed.^[23]^

To our knowledge, no attempts at utilizing the acetylacetone substituent in condensation reactions, such as the formation of pyrazoles or thiosemicarbazones, have been described thus far. Other compounds based on the same methodology have been reported by Pascal et al., who used malononitrile as a carbon nucleophile, and by Nagao et al., who used barbituric acid in a similar way.[^8b^, ^8c^] The fourth carbon nucleophile that has been used, albeit only in patents which do not describe the synthesis or the photophysical properties, is cyanide.^[19a]^ While it seems rather obvious to use this readily available, highly nucleophilic pseudo‐halide, it is completely missing from the scientific literature and discussion. A reason for this may be a side‐reaction in which the cyanine attacks the bridgehead carbon at either end of the polymethine chain next to one of the nitrogen centers.^[24]^ This reactivity has been exploited in the design of ratiometric probes for the quantification of cyanide.[^19b^, ^24^] Štacková et al. published a *meso*‐nitrile derivative as one of the structures obtained through ring‐opening of Zincke salts.^[10a]^ Although their structures do not feature a rigidified polymethine chain, *meso*‐CN substitution in their scaffold leads to a red‐shift of absorption and emission maxima as compared to most other derivatives.

**Scheme 4:**
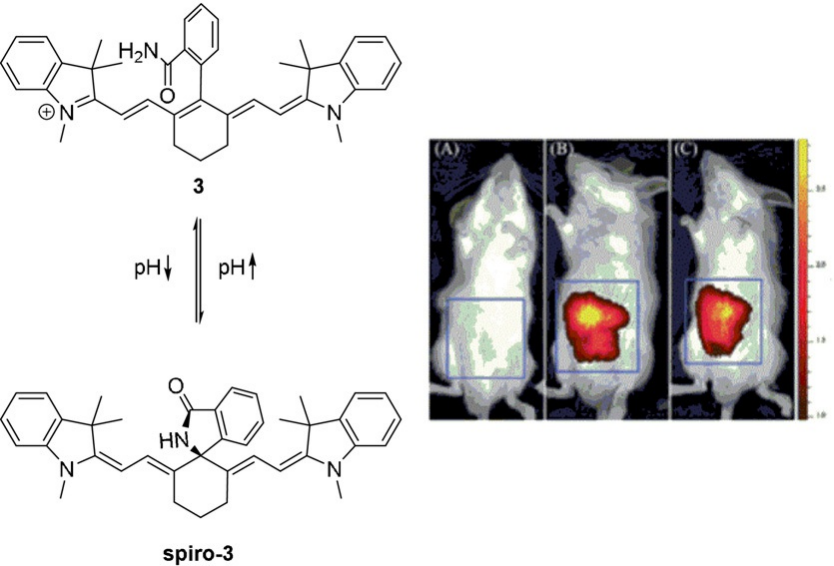
*Meso*‐spiro reactivity of a tricarbocyanine dye. The authors described the use of **3** as an imaging agent for acidic microenvironments, often associated with inflammation. From left to right: control, LPS (lipopolysaccharides, provoke acute inflammatory response) with **3**, and **3**. Images used with permission from Ref. [26].

A larger number of accounts describe the introduction of carbon‐based substituents by means of cross‐coupling methods.[^11a^, ^11c^, ^18d^, ^25^] Suzuki–Miyaura reactions typically employing palladium(0) complexes, such as Pd(PPh_3_)_4_, in conjunction with the appropriate boronic acid precursor have been described.[^11c^, ^25^] The reaction is typically performed in water or aqueous solvent mixtures, such as water/dioxane mixtures.[^11a^, ^11c^, ^18d^, ^25^, ^26^] Apart from the mentioned stability improvements such *meso*‐carbon dyes offer, they may also be used to produce probes which, dependent on the pH, reversibly form heterocyclic spiro derivatives (Scheme 4). At low pH, compound **3** is fluorescent and the rings are not fused, while at higher pH the nucleophilic *ortho*‐substituent attacks the *meso*‐position, forming a non‐fluorescent spirane.^[26]^

Alternatively, Sonogashira cross‐couplings may be performed to give *meso*‐alkyne derivatives, which tend to be red‐shifted with respect to the parent dye.^[11a]^ With simple aryl systems coupled at the *meso*‐position, a hypsochromic shift of ca. 20 nm is observed. However, the Stokes shift of such dyes was often reported to be slightly smaller (10–15 nm) than that of the parent dye. The resulting dyes are reported to possess excellent chemical and optical stability in vitro.^[18d]^ However, even in highly sulfonated cyanine dyes, self‐aggregation is observed in *meso*‐phenyl derivatives, potentially limiting their usefulness in vitro and in vivo.^[11a]^

The synthesis of a sterically shielded non‐rigid cyanine dye with remarkable properties through the ring‐opening of a Zincke salt was also reported alongside its conjugation to cyclic‐RGD peptide and (goat) IgG; this demonstrated the effect of the steric shielding on the photophysical properties of the dye.^[10b]^ Most noticeably, the sterically shielded derivative did not show obvious signs of aggregation on the protein surface, unlike other peptide or antibody–cyanine conjugates, which in many cases show hypsochromic shifts and signs of H‐aggregate formation.[^10b^, ^27^] It could be conceived that cyanine‐dye‐based rotaxanes^[28]^ conjugated to antibodies may behave in a similar fashion and limit aggregation and aggregation‐caused quenching.

### 3.2 Introduction of Nitrogen Substituents

**Scheme 5:**
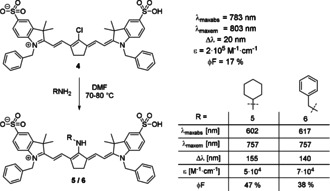
Synthesis of *meso*‐amine tricarbocyanine dyes by Peng et al. Photophysical properties measured in water at a concentration of 1 μm. The fluorescence quantum yields were determined in reference to IR‐125 (for **4**) and rhodamine B (for **5** and **6**).^[18c]^

The introduction of nitrogen substituents in the *meso*‐position is a straightforward way to introduce new properties and functionalities.^[6c]^ While only few examples of dyes with an unsubstituted amine in the *meso*‐position exist, numerous accounts have been published on the synthesis of substituted amines. To obtain a *meso*‐NH_2_‐functionalized cyanine dye, a two‐step procedure is necessary. Some patents claim to use phthalimide as a nucleophile and reduce the intermediate product.^[29]^ Alternatively, *meso*‐NH_2_ dyes may be accessible through synthesis of the corresponding *meso*‐azide derivative as an intermediate product and subsequent reduction with H_2_S, as reported by Yu et al.^[20]^ While aminocyanine dyes obtained from the reaction with substituted amines were first prepared in the second half of the 20^th^ century, one of the first investigations into their peculiar properties was undertaken by Gray et al. in 1996.[^1a^, ^30^] For several years, only few publications concerning aminocyanine dyes appeared until Peng et al. synthesized two new derivatives in 2005 and noticed their large Stokes shift (Scheme 5, dyes **5** and **6**).^[18c]^ Amination with primary amines is typically achieved under mild conditions in polar, aprotic solvents.[^17b^, ^18c^, ^31^] The resulting aminocyanine dyes show an intense blue‐shift (up to 180 nm) compared to their *meso*‐chloride counterparts, as well as a significantly larger Stokes shift (in some cases larger than 100 nm).[^8b^, ^18c^] In general, the changes observed due to amination can be understood as a perturbation of the conjugated system due to the interaction of the lone pair of nitrogen with the conjugated polymethine system.

**Scheme 6:**
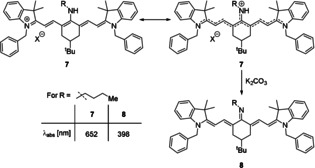
Resonance structures of aminocyanine dyes (top), and proposed structure after treatment with base. Absorption and emission values according to Pascal et al.^[8b]^

As such, a resonance structure in which the central position can tautomerize, giving either an enamine or an imine, is the most straightforward way to visualize the electronic situation (Scheme 6). In primary amines this correlates to stronger blue‐shifts in electron‐rich structures and weaker blue‐shifts in electron‐poor substituents, while amino groups with two alkyl substituents lead to weaker blue‐shifts, presumably due to the increased steric demand.[^8b^, ^13b^] If *meso*‐amine dyes are treated with a base, such as potassium carbonate, an even greater hypsochromic shift is observed, which would correspond to an even more pronounced perturbation of the conjugated system and the emergence of an actual imine bond (Scheme 6, dye **8**).^[8b]^

It was suggested that the unexpectedly large Stokes shifts observed in these dyes were the result of an excited state intramolecular charge transfer (ICT).^[18c]^ However, it seems that this is related to a drastic change of the overall electronic structure, which resembles an imine‐type bonding in the ground and shifts towards an amine bond in the photoexcited state.[^30^, ^32^] In addition to the character of the C−N bond in the *meso*‐position, symmetry breaking in the ground state was recently suggested to cause the broad absorption bands and large Stokes shifts.^[33]^

While the *meso*‐nitrogen becomes more electron rich, steric hindrance increases and inhibits an effective overlap between the polymethine π‐system and the nitrogen's lone pair, effectively leading to weaker blue‐shifts than their counterparts with a lower degree of substitution.

According to Gray et al., the isomerization of *meso*‐amine cyanine dyes leads to low quantum yields due to the existence of both, a non‐fluorescent and a fluorescent form, depending on the orientation of the amine substituent.^[30]^ Cao et al. investigated the influence of the position of the *meso* substituent on the photophysical properties in silico.^[32]^ They found that an amino group at an odd chain position (as counted from the indole) acts as an electron donor in the excited state. However, if it is situated at an even position within the polymethine chain, it acts as an electron acceptor instead. The energy barrier for rotation of the NH_2_ group at an even position was found to be significantly larger (7.09 eV at the center of the chain, 0.1 and 0.06 eV at odd positions).^[32]^

Considering these findings and the reports by Gray et al., the high quantum yields reported by some authors seem disproportionate and may be the result of using unsuitable fluorophores as reference.[^18c^, ^31a^, ^34^] Although some variability may be caused by using different cyanine backbones, such large differences are not to be expected. However, due to the large Stokes shift exhibited by aminocyanines (up to ca. 150 nm), relative measurements may lead to inaccurate determinations of fluorescence quantum yields. Furthermore, such a large Stokes shift may lead to relative overestimation of quantum yield due to a lack of reabsorption of the emitted light. This is especially troublesome if the standard used in the relative measurement possesses small Stokes shifts. Literature values for quantum yields range from low single‐digit values to almost 50 %.[^18c^, ^31a^, ^34^]

It has been reported that upon protonation of aminocyanine dyes featuring diamino substituents, such as piperazine, an intense bathochromic shift has been observed: this corresponded to the formation of an ammonium group. This effectively halts the perturbation of the polymethine system[^17b^, ^17d^] and leaves the electron‐withdrawing inductive effect, causing a hypsochromic shift, relative to the *meso*‐Cl dye, as would be expected from ammonium substituents.[^17b^, ^17d^] There are accounts of blue‐shifts upon protonation, especially in amine substituents derived from primary amines and monoamines.[^18c^, ^31a^] This was suggested to occur as the result of an irreversible protonation of the polymethine system.^[35]^ The effect of protonation on quantum yield has not yet been fully elucidated for *meso*‐diamine substituents. It has been reported that the extent to which protonation affects the photophysical characteristics, such as quantum yields, varies greatly within the series, thus further studies are necessary.[^17b^, ^17d^]

**Scheme 7:**
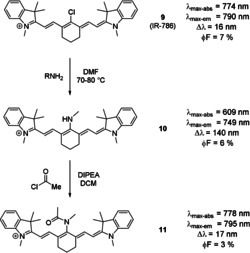
Synthesis of *meso*‐amine tricarbocyanine dye and subsequent amide formation using an acid chloride. Photophysical properties next to each dye.^[31a]^ Counterions omitted for clarity.

The formation of amide bonds from a *meso*‐nitrogen substituent leads, much like protonation in some dyes, to an intense red‐shift, which almost re‐establishes the original optical properties of the dye (Scheme 7, dye **11**).[^17b^, ^31a^, ^36^] This is a result of the involvement of the nitrogen's lone pair in a resonance structure with the carbonyl. Likewise, aromatic amines in the *meso*‐position lead to significantly smaller differences in terms of absorption maximum, due to the electron donation into the aryl system.[^8b^, ^13b^]

**Scheme 8:**
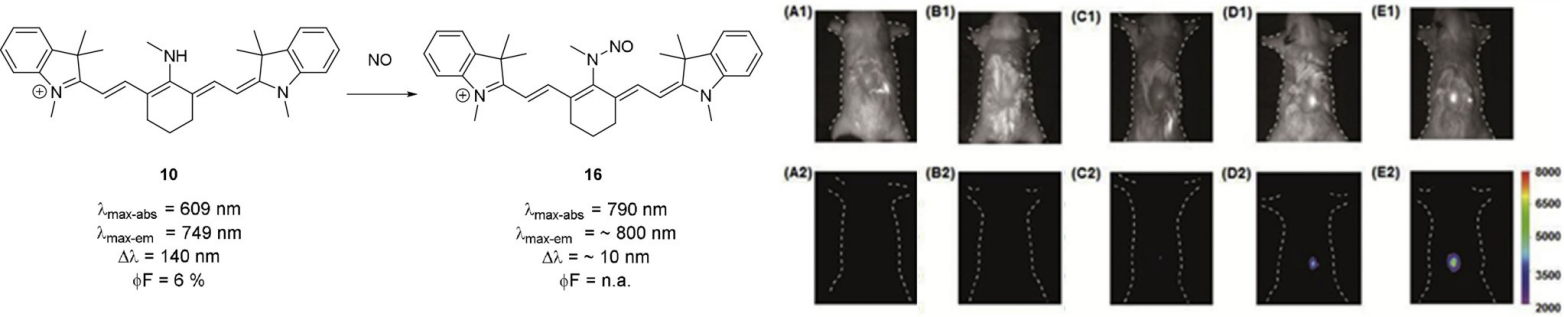
Top row: use of **10** as a switch‐on probe for nitric oxide in vivo. Counterions omitted for clarity. Bottom row: fluorescence imaging of living mice after subcutaneous injection of A) saline, B) NaClO_3_, C) H_2_O_2_, D) DEA‐NONOate sodium salt (a nitric oxide donor), and E) LPS (as a model for inflammation). Three hours after injection, **10** was injected subcutaneously. Significant increase of signal intensity can be seen with DEA‐NONOate and LPS. Image used with permission from Ref. [37].

Dye **10** (Scheme 8) has the potential to act as a switch‐on probe for nitric oxide.^[37]^ Upon addition of NO to the *meso*‐nitrogen, the imine‐type resonance structure is suppressed, and the fluorescence intensity increases significantly (Scheme 8). Apart from these structurally simple examples, various aminocyanine dyes have been synthesized, bearing functional moieties, targeting molecules, or groups for further conjugation in the *meso*‐position. Examples include a sphingosine derivative (an amino alcohol, and a primary part of sphingolipids, which make up parts of the cell membrane),^[34]^ photocleavable prodrugs such as tamoxifen‐like compounds,^[38]^ (PEG)ylated derivatives such as those reported by Lu et al.,^[39]^ ratiometric probes for nitroreductase,^[40]^ and agents for photodynamic therapy, as reported by Jiao et al.^[41]^ Alternative strategies also include pH cleavable prodrugs, as reported by Xing et al.,^[42]^ and pH activated agents for photodynamic therapy.^[43]^

A limitation in the use of aminocyanines may be their lack of chemical stability, as solutions in serum or cell culture medium deteriorate over time.^[13a]^ Likewise, they are prone to photobleaching compared to their respective parent compounds or other *meso*‐substituents. This is likely caused by the increased electron density in the backbone, effectively increasing reactivity towards reactive oxygen species.

**Scheme 9:**
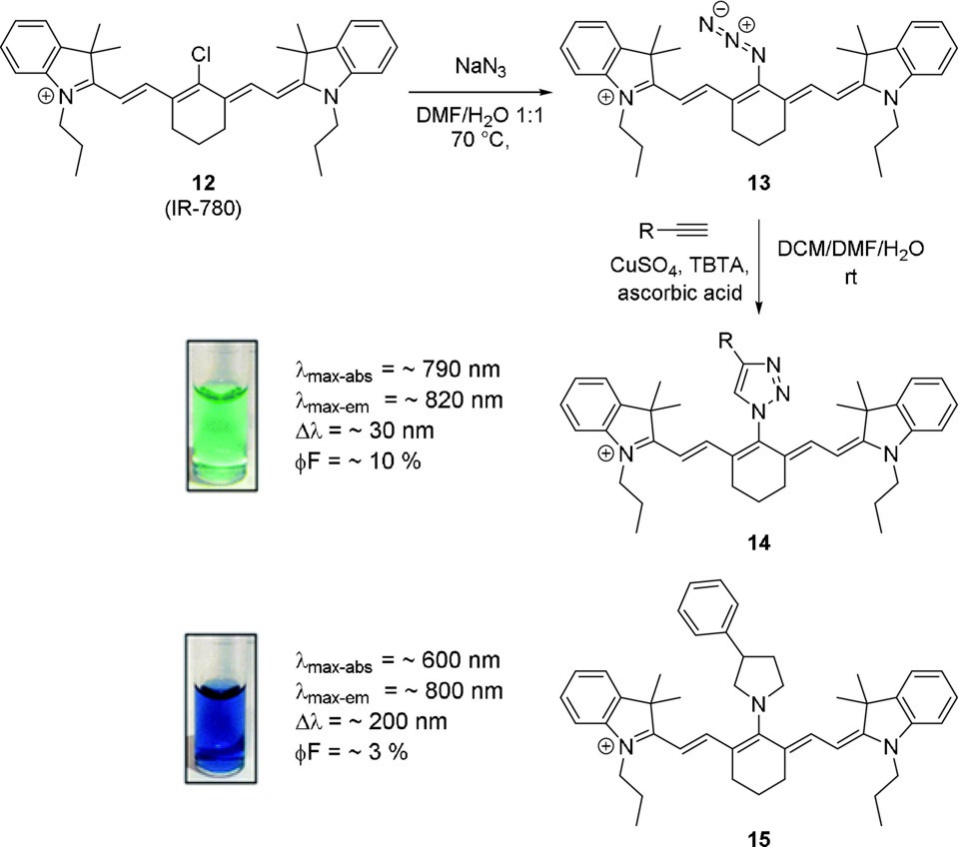
Synthesis of *N*‐triazole‐functionalized cyanine dyes and comparison with a secondary amine substituent. Image used with permission from Ref. [18a]. Counterions omitted for clarity.

One way to circumvent the limited photostability of such aminocyanine dyes is the use of *N*‐triazole‐substituted cyanine dyes, as described by Mellanby et al. (Scheme 9).^[18a]^ These dyes showed improved photophysical properties as compared to the parent dye, such as (slightly) larger Stokes shift, greater photostability, and higher molecular brightness. It has been suggested that the reason for this was the electron donation into the triazole, rather than into the polymethine system.

In many cases, the library of triazole‐functionalized dyes possessed higher molecular brightness than the original *meso*‐Cl dye. Utilizing an asymmetric derivative of the triazole‐functionalized dye featuring a carboxylic acid moiety activated by formation of an NHS‐ester, Mellanby et al. reported the labeling of T‐cells and performed non‐invasive fluorescence imaging in mice.^[18a]^ More recently, a number of bifunctional probes featuring three triazole handles was published.^[3b]^ Using absolute quantum yield measurements, the authors determined a value of 3.22 % in PBS for a (PEG)ylated derivative (as compared to 1.90 % for ICG).^[3b]^

Despite the significant amount of research results published regarding aminocyanine dyes,[^17d^, ^18c^, ^30^, ^32^, ^41^] several fundamental considerations remain a matter of debate. For example, the effect of the amine substituent on quantum yield remains to be definitively answered, ideally by measurements of fluorescence quantum yield with an integrating sphere. If the amine is prevented from perturbing the conjugated system, it remains to be clarified whether the quantum yield increases or decreases. Further investigations into the role of solvents and concentration would likewise be of interest.

### 3.3 Introduction of Oxygen Substituents

**Scheme 10:**
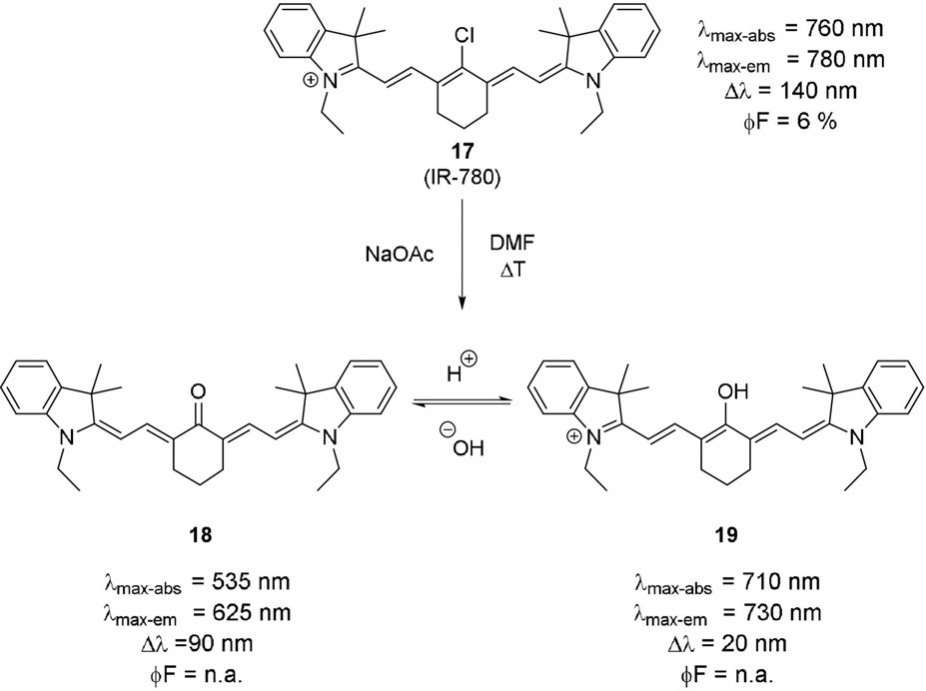
Synthesis of oxygenated derivatives of rigid tricarbocyanine dyes and pH‐induced keto–enol equilibrium. Counterions were omitted for clarity.

The introduction of oxygen in the *meso*‐position can be accomplished in two different ways. The *meso*‐Cl fluorophore may be reacted with a nucleophilic oxygen compound (e.g. a phenol in the presence of base).[^6c^, ^44^] This reaction has been reported to not proceed well with simple, non‐aromatic alcohols, presumably because the reaction proceeds via an S_RN_1‐type mechanism.[^6c^, ^45^] Nevertheless, non‐aromatic *meso*‐ethers may be obtained through Smiles rearrangements.^[45]^ The latter methodology has been applied to synthesize both, probes for imaging of the ureter^[46]^ and antibody–dye conjugates to study the effect of charge localization on the in vivo properties of the resulting conjugates.^[47]^ Alternatively, oxygen, in the form of an enol or ketone, may be introduced under oxidative conditions. This may be accomplished by heating the *meso*‐Cl dye in a basic solution of dimethylformamide^[48]^ or through oxidation with a catalytic amount of *N*‐hydroxysuccinimide in alkaline solution.^[49]^ Both reactions are mild oxidative methods and as such indicate the high reactivity of the C−Cl bond in these molecules. The photophysical behavior of the probes obtained by these methods can best be described by assuming a keto–enol equilibrium (not tautomerization) in protic solvents (Scheme 10, **18** and **19**).[^48^, ^50^] The fact that the underlying isomerization process is not a tautomerization is evident due to the positive solvatochromism observed under pH‐neutral conditions, suggesting an uncharged ground state.[^49a^, ^51^] Similar to the situation described above for primary amine substituents, these simple oxocyanines show bathochromic shifts of ca. 200–300 nm and Stokes shifts approaching 100 nm in the keto form.[^48^, ^52^] It is noteworthy that the Stokes shifts of these dyes are significantly larger (about 30 to 50 nm) in protic solvents, which may be related to hydrogen bonding between the ketone and polarized hydrogen atoms.[^49a^, ^51^] Additionally, the fluorescence quantum yield in protic solvents is significantly higher.^[51]^

Upon protonation, the dye is found in its enolic form. This change is accompanied by a significant hypsochromic shift, as the conjugation between the nitrogen centers is re‐established. Compared to their *meso*‐Cl counterparts, the enol dye still shows a significant hypsochromic shift (ca. 50–100 nm). Unfortunately, fluorescence properties of these oxocyanines dyes have not been investigated in detail. Zheng et al. and Pascal et al. provided quantum yields.[^49a^, ^51^] Their values are highly solvent‐dependent, and significantly higher (in protic solvents) than the parent dye's quantum yield.

Much like aminocyanine dyes, *meso*‐oxocyanines exhibit the expected reactivity, that is, they react as nucleophiles themselves, and can be reacted with acid halides[^48^, ^50^, ^53^] and sulfonyl halides.^[54]^ Rates of photobleaching are highly dependent on the protonation state of the oxocyanines. In the keto form, photobleaching occurs at a much lower rate compared to the parent compound. In the enol form, photobleaching occurs at a much higher rate.^[49a]^

**Scheme 11:**
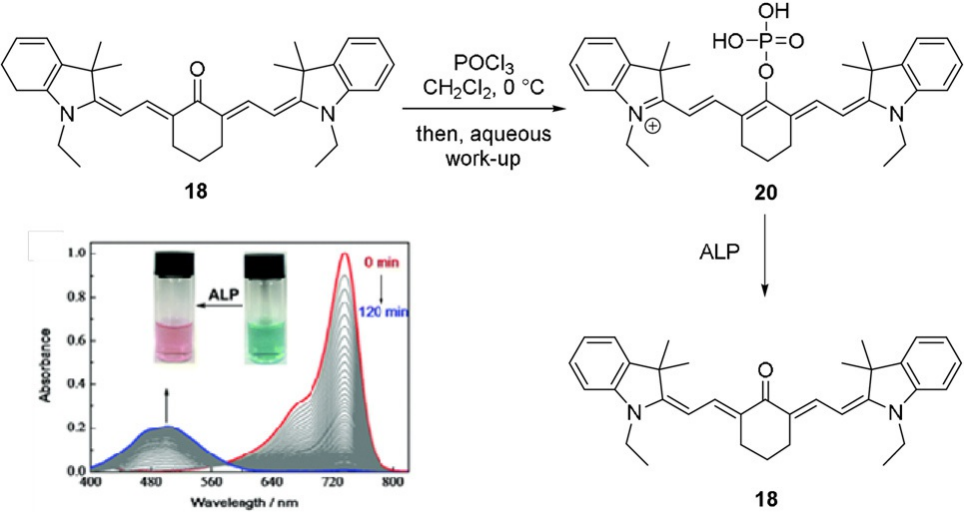
Derivatization of oxocyanine dyes by reaction with phosphoryl chloride and subsequent cleavage of the phosphorous−oxygen bond by alkaline phosphatase (ALP). Image adapted from Ref. [55] with permission. Counterions omitted for clarity.

Reactions of oxocyanine dyes with phosphorous reagents have, to our knowledge, only been reported by Zhang et al., who reacted dye **18** with phosphoryl chloride (Scheme 11).^[55]^ It may be considered a proof of concept for the design of turn‐on ratiometric probes, as the phosphorylated probe's photophysical characteristics are similar to that of the parent dye, while upon dephosphorylation by alkaline phosphatase, a change back to the absorption characteristics of **18** is observed.

**Scheme 12:**
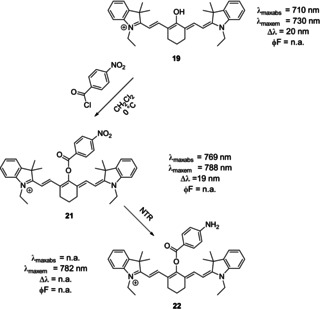
Derivatization of oxocyanines dyes by reaction with acid chlorides. Upon enzymatic reduction of the *p*‐nitro substituent of **21** by NTR, fluorescence intensity increased more than 100‐fold (**22**).^[57]^ Counterions omitted for clarity.

Some authors employed similar methodologies to establish fluorescent probes for the detection of hydrogen sulfide and hydrazine.[^50^, ^56^] Li et al. synthesized a fluorescent switch‐on probe for the detection of nitroreductase (NTR) (Scheme 12), an enzyme which is overexpressed in many hypoxic tumors.^[57]^ A caveat in using probe **21** is that slow side‐reactions with cysteine and glutathione may occur.[^50^, ^58^]

Likewise, Hu et al. demonstrated that esters in the *meso*‐position can be cleaved under reductive conditions.^[53]^ They utilized this reactivity to prepare ratiometric probes for the detection of hydrazine.

Furthermore, *meso*‐ethers, and especially *meso*‐phenyl ethers, have been found to be kinetically unstable towards many endogenous thio‐nucleophiles, including cysteine and glutathione.^[44]^ Nevertheless, the IR 800CW conjugates have been successfully used in vivo. The extent to which the formation of a glutathione adduct of the IR 800CW dye influences its photophysical properties in vivo remains a matter of investigation.^[25]^

### 3.4 Introduction of Thio Substituents

**Scheme 13:**
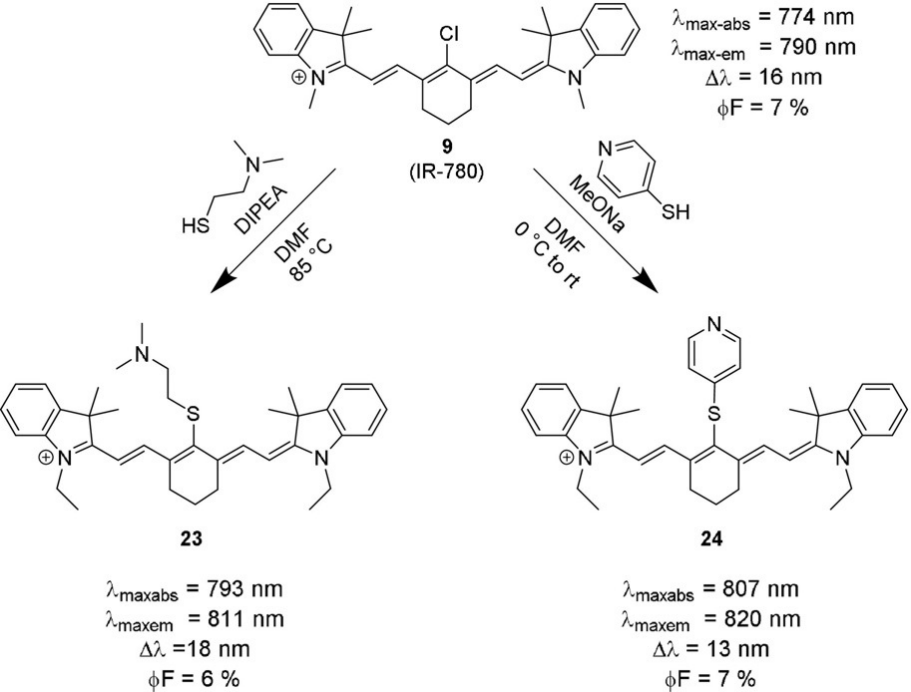
Reaction of *meso*‐Cl dye **9** with thio‐nucleophiles gives *meso*‐thioethers with both aliphatic (**23**) and aromatic substituents (**24**).^[13b]^ Counterions omitted for clarity.

The formation and biological fate of thiocyanine dyes are of particular interest for many biochemical and medicinal applications, as many endogenous species contain highly nucleophilic thio substituents, and several enzymes catalyze the formation of thiols, or reactions involving them. *Meso*‐arylethers may undergo nucleophilic substitution under physiological conditions and form *meso*‐thio derivatives. As fluorescent labels for proteins, these functional NIR dyes may allow for straightforward detection and quantification. In terms of photophysical properties, they typically show a small bathochromic shift compared to the parent dye. In thioethers with an aromatic substituent, the bathochromic shift is larger than in thioethers with non‐aromatic substituents.[^13b^, ^59^] Photostability is similar to the original *meso*‐Cl derivatives, and mostly comparable to the *meso*‐aryl and *meso*‐alkyl ethers.^[13b]^ Not much is known about their chemical stability. Although some authors suggested that thioethers may undergo further reactions or rearrangements,[^44^, ^45^] several conjugates were found to be kinetically stable.[^18b^, ^45^] The stability may be related to steric hindrance or other, yet unelucidated factors. The quantum yield of aromatic thioethers seems to be smaller than those of arylethers, but larger than those of aryl amines.^[13a]^ Their molar absorptivity and molecular brightness in PBS and FBS were reported to be lower than that of the respective parent dye or aryl ethers for both aliphatic and aromatic thioethers (Scheme 13).^[13a]^ No turn‐on/turn‐off reactivity has been described to our knowledge, which is in stark contrast to the amino‐ and oxocyanine dyes discussed above. Nevertheless, they may be useful ratiometric probes for certain biomarkers or enzyme activity. Several such probes based on ICT‐type mechanisms have been described, for instance for *N*‐acetyltransferase^[60]^ or nitroreductase.^[57]^

Like the *meso*‐arylethers, *meso*‐Cl dyes were found to undergo nucleophilic substitution with a range of endogenous thiol‐based nucleophiles.[^11b^, ^18b^, ^27b^, ^61^] This reactivity may be used to directly label peptides and proteins and could, by extension, be used to directly label monoclonal antibodies, or fragments thereof. It has also been suggested to influence the pharmacokinetics and to be the reason for the observed tumor selectivity and retention of *meso*‐Cl dyes, through formation of covalent albumin conjugates.^[27b]^

The formation of dye–albumin conjugates appears to proceed in two steps. First, a non‐covalent aggregate is formed, leading to a bathochromic shift. Then, the dye reacts with a free thiol, forming a covalent bond, which leads to a small hypsochromic shift.^[27b]^ Overall, the dye–albumin conjugate is red‐shifted relative to the *meso*‐Cl dye, consistent with the formation of a thiocyanine. It has been suggested that the observed selectivity and strong retention of such tumor‐seeking *meso*‐Cl dyes is caused by a combination of overexpression of albumin receptors on tumor cells and the EPR effect.^[27b]^ Other fluorophore–albumin conjugates have been reported to behave similarly and have been investigated for their use as imaging agents in the treatment of various cancers.^[62]^ Lin et al. used the reactivity of a *meso*‐Cl dye to label vimentin (a structural protein) and several other proteins.^[11b]^ It was found that amounts as low as 1 ng substrate are detectable on a gel‐electrophoresis imaging device. Canovas et al. applied an analogous approach in the labeling of peptides, albumin, and the antigen‐binding fragment of pertuzumab (Fab, for the treatment of HER2 positive breast cancer).^[18b]^

Figure 1 shows the structure of a cyanine sulfonyl derivative (CCDC‐272535). Although the crystal structure has been deposited, no literature is available detailing its synthesis or optical properties. A possible way could be the oxidation of the corresponding *meso*‐thiophenyl ether with a suitable oxidizing agent like OXONE® or periodate.

## 4 Conclusion and Outlook

In the development of heptamethine cyanine dyes, some classes of substituents have, historically, been difficult to access. Recently, new synthetic methodologies which may overcome these barriers were developed and may help resolve some of the current limitations. Even if these methods do not yield rigid dyes, they offer access to new *meso*‐substituents and substitution patterns. These could offer novel reactivities or improved photophysical characteristics. Some derivatives that could be of interest and were previously not accessible, or difficult to synthesize, include *meso*‐fluoride dyes, as well as some pseudohalide derivatives, such as *meso*‐nitrile or *meso*‐thiocyanates. The photostability of some derivatives remains an issue, and additional work to further elucidate the causes of the observed differences is needed. The discrepancies encountered in the literature call for the establishment of standardized procedures so the properties of these dyes can be compared and evaluated in a rational and robust manner. This would help pave the way towards the development of the full potential of tricarbocyanine dyes. More recently, various authors suggested and demonstrated the use of the off‐peak tail emission of such dyes, or albumin chaperoned dye aggregates, which extends into the second NIR window (NIR‐II, 1000–1700 nm).^[63]^ The use of compounds already established for NIR‐I imaging could significantly ease the establishment of imaging in the NIR‐II window.[^63^, ^64^] Likewise, further development of cyanine dyes for applications such as PDT is of great interest, due to the penetration depth of light in the NIR window and their high molar absorptivity.[^3b^, ^41^, ^43^, ^65^]

Notwithstanding the extensive synthetic advances achieved, and some of the commercial opportunities presented regarding the production of *meso*‐substituted cyanine dyes,^[66]^ a general transition as everyday probes either in in vivo settings or in the clinic remains to be seen. This is in contrast to the promising photophysical properties, the synthetic simplicity of the introduction of substituents in *meso* cyanine dyes, and the sheer amount of possible applications and probes that can be realized.

The success of IR 800CW, currently in clinical trials for the fluorescence‐guided surgery of several cancers (e.g. of the brain, head and neck, esophageal, breast, lung, pancreatic, kidney, and colorectal cancer),^[67]^ is a good indicator of the progress that has been made and the general usefulness of such fluorescent probes.

## Conflict of interest

The authors declare no conflict of interest.

## Biographical Information

*Rüdiger M. Exner is a PhD student at the University of Bath. He obtained his B.Sc. and M.Sc. at the University of Wuppertal with theses on the chemistry of dodecaborates. While pursuing his master's degree he was awarded an Erasmus+ grant and spent several months at the University of Oulu. He then joined the Max Planck Institute for Coal Research for a short research stay. He currently develops multimodal imaging probes for the recognition of prostate cancer biomarkers*.

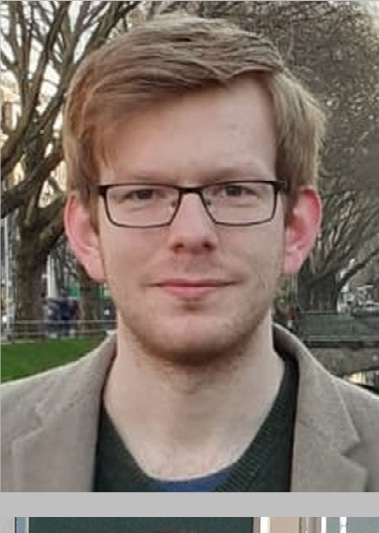

## Biographical Information

*Fernando Cortezon‐Tamarit is a Research Fellow at the University of Bath developing molecular probes for the imaging of hypoxia under the O2SENSE ERC project in the team of Prof. S. I. Pascu. During his PhD he carried out research in imaging probes for prostate cancer as part of the Marie Curie ITN PROSENSE. The targeted imaging probes synthesized during his recent experience include metal complexes with a variety of ligands, organic fluorophores for sensing, and carbon nanomaterials*.

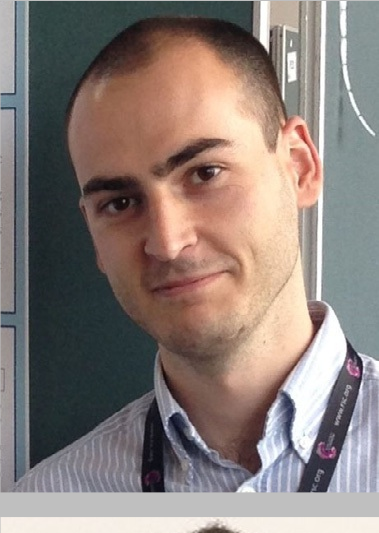

## Biographical Information

*Sofia Pascu is Professor of Bioinorganic and Materials Chemistry at the University of Bath. She held a RS URF award (2005–2015) and is an ERC Consolidator grantee (2014–2020). After her DPhil (Chemistry) from Balliol College, Oxford and a PDRA at the Inorganic Chemistry Laboratory University of Oxford working with Prof. M. L. H. Green FRS (1997–2003) she was an EPSRC PDRA at the Chemistry Department, University of Cambridge (2003–2005) with Prof. J. Sanders FRS and Prof. A. Holmes FRS. Her research interest focuses on molecules and materials with applications in catalysis, sensing, and imaging and their biophysical characterization*.